# Medical and pharmacy students’ perspectives of remote synchronous interprofessional education sessions

**DOI:** 10.1186/s12909-022-03675-2

**Published:** 2022-08-10

**Authors:** Hend E. Abdelhakim, Louise Brown, Lizzie Mills, Anika Ahmad, James Hammell, Douglas G. J. McKechnie, Tin Wai Terry Ng, Rebecca Lever, Cate Whittlesea, Joe Rosenthal, Mine Orlu

**Affiliations:** 1 UCL School of Pharmacy, 29-39 Brunswick Square, London, WC1N 1AX UK; 2 UCL Research Department of Primary Care & Population Health, Royal Free Campus, Rowland Hill Street, London, NW3 2PF UK

**Keywords:** Interprofessional Education, Remote learning, Pharmacy, Medicine

## Abstract

**Background:**

Interprofessional education (IPE) at university level is an essential component of undergraduate healthcare curricula, as well as being a requirement of many associated regulatory bodies. In this study, the perception of pharmacy and medical students’ of remote IPE was evaluated.

**Methods:**

A series of IPE sessions took place via Zoom and students’ feedback was collected after each session. Both qualitative and quantitative data were collected and analysed.

**Results:**

72% (23/32) of medical students strongly agreed that the sessions had helped to improve their appreciation of the role of pharmacists, whereas 37% (22/59) of pharmacy students strongly agreed, reporting a median response of ‘somewhat agreeing’, that their appreciation of the role of general practitioners had improved. This difference was found to be statistically significant (*p* = 0.0143). Amongst students who responded, 55% (53/97) identified remote teaching as their preferred mode of delivery for an IPE session.

**Conclusions:**

The survey demonstrated that the students valued the development of their prescribing skills as well as the ancillary skills gained, such as communication and teamwork. Remote IPE can be a practical means of improving medical and pharmacy students’ understanding of each other’s professional roles, as well as improving the skills required for prescribing.

## Introduction

Interprofessional education (IPE) defines the process whereby two or more professions learn about, from, and with each other to enable effective collaboration and improve health outcomes [1]. Traditionally, many healthcare IPE activities have involved sending students on shared clinical placements, where it is assumed they will interact with other healthcare professionals, however, these approaches vary across universities [2]. Although this provides valuable experience there has been little emphasis on interaction with student peers on different healthcare courses [3]. Promoting IPE at undergraduate level—supports the National Health Service (NHS) Long Term Plan [4], which includes the aim of bringing together different healthcare professionals to co-ordinate better patient care, changing the health service for a more engaged relationship with patients [5]. The NHS Long Term Plan also launched Primary Care Networks [6], for whom interprofessional working between general practitioners (GPs) and pharmacists is a key component. In addition, in the English NHS, Integrated Care Systems are now building new partnerships between healthcare organisations to improve interprofessional collaborations, and thereby patient outcomes [7]. A mapping study of five United Kingdom (UK) health professions’ regulatory bodies (medicine, pharmacy, dentists, nursing & midwifery and allied healthcare professions) found that seven themes were identified as common learning outcomes for students. The themes identified were: knowledge for practice; skills for practice; patient-centred approach; ethical approach to practice; continuing professional development; team-working; and professionalism [3].

IPE is a requirement for pharmacy and medical programmes to be reaccredited as set by their respective regulatory bodies, the General Pharmaceutical Council (GPhC) and the General Medical Council (GMC) [2]. IPE allows students to learn about the work of other practitioners, which in turn improves patient outcomes by training future healthcare workforce and creating collaborative practice [1]. The specific needs to implement IPE activities include co-ordination of timetable requirements of participating degree programmes and general cross-programme organisational support [8]. Olenick et al. found that having an IPE lead overseeing the whole team facilitated the smooth running of the IPE sessions and this is recommended when planning future activities [9].

In a recent review [10], IPE activities across UK pharmacy schools were found to be inconsistent, but most schools did run some form of IPE session on average once a year, usually for the equivalent of one full day. Globally, the variation is also observed among the required student competencies among the Unites States (US) healthcare degree programmes hence professions are encouraged to harmonise their accreditation standards in their IPE programmes. In the US and Canada, national efforts forge consensus via shared competency frameworks such as IPE Collaborative (IPEC) and Canadian Interprofessional Health Collaborative (CIHC) [11–13]. In the UK, although different universities adopt different IPE styles, a formal approach, known as the Leicester Model, has been recommended [14]. The model outlines learning outcomes to focus on knowledge and attitudes of team working, a practical understanding of multi-disciplinary service delivery and an appreciation of the patient at the centre of service delivery. It is to be noted that this model has been developed for face-to-face activities, however, the themes derived from it can be used to organise a remote IPE session. Remote IPE is an area of recent interest with potential to overcome logistical, geographical and organisational challenges previously identified as being a barrier to IPE [1]. Distance learning was historically adopted for various educational interventions of collaborative learning programmes [15, 16]. Virtual IPE has been originally designed to accommodate large student cohorts from multiple disciplines and timetable requirements and recently utilised in the delivery of education during the Covid-19 pandemic [17].

The general IPE curriculum includes workshop-based sessions with the theme of prescribing, for Pharmacy and Medical School students at University College London (UCL). In this study, remote IPE sessions were organised for fourth-year pharmacy students and fifth-year medical students. The educational content for IPE in prescribing in primary care sessions was originally developed for face to face teaching delivery in 2019 and shifted to the remote setting due to the Covid-19 outbreak. Published literature showed the lessons learnt from the innovative trials of emergency remote teaching during the Covid-19 pandemic and suggested exploring the virtual IPE models further [18–20]. In order to maintain the effective teaching delivery and student engagement during the remote sessions, video communication and online polling tools were added as an interactive component to the IPE sessions at UCL School of Pharmacy.

In this study, the aim of the IPE sessions was to improve the respective awareness of the students of both professions and to educate them about common themes, in particular prescribing in primary care and dispensing legislation of medicines. The results presented in this study focus on the learners’ feedback and their evaluation of the sessions. The overall aim of this paper was to explore student’s perspectives towards IPE education and to use that information to improve the design of these sessions in the future.

## Methods

This research study has been covered under the UCL Ethics Application: 12,143/003: Education Research Projects at the UCL School of Pharmacy. All participants were invited to complete the voluntary IPE evaluation survey at the end of each IPE session via providing them the survey link and Quick Recognition (QR) code.

### Study design

The design of the study was based on gathering both quantitative and qualitative data to infer participants’ attitudes towards the IPE session. All students that took part in the IPE activity were invited to complete the survey at the end of the sessions. Consent in taking part in the survey was implied when the student actually completed the questionnaire; there were no explicit consent forms. Data gathered did not contain any identifying information about participants and the survey was stored on the Qualtrics software system.

### Setting

The IPE initiative between the UCL School of Pharmacy and UCL Medical School was organised via an IPE lead. Information on the workshop was made available to students prior to the sessions via the University’s Virtual Learning Environment (Moodle). IPE curriculum aims for explaining the importance of prescribing medicines safely, appropriately, effectively and economically in primary care; recognising the risks of prescribing errors; describing the roles, responsibilities and approaches of pharmacists and general practitioners whilst dealing with clinical problems and the ways in which they communicate and collaborate to improve patient outcomes. IPE workshops were planned and designed with equal contribution from the teaching staff of UCL Medical and Pharmacy Schools. Case studies of IPE workshops were developed to enable both healthcare professions` students to learn with and from each other by selecting topics requiring knowledge of GPs and pharmacists in primary care. The sessions were planned to maximise the interactive discussions between mixed groups of medical and pharmacy school students. In order to maintain the student engagement, online learning tools were implemented in the delivery of remote IPE sessions.

A technical lead was accessible if their help was required. Students were assessed through a reflection in their portfolio.

### Content

IPE in prescribing in primary care sessions were compulsory for the entire cohorts of fourth year Master of Pharmacy (MPharm) and fifth year Bachelor of Medicine and Bachelor of Surgery (MBBS) Programme students at UCL. Both student groups were final year students in their respective Programmes and had prior inter-professional learning experience in the field of safe prescribing. Students were not asked to submit compulsory work prior to attendance to IPE workshops and prework was only offered to better prepare them to the IPE subject field.

The workshops consisted of two clinical case studies focusing on prescribing in primary care. Teaching was co-facilitated by GPs and pharmacists who worked with students through these pre-prepared clinical cases with practical exercises including how to identify and manage polypharmacy, drug interactions, prescribing for pain and prescribing controlled drugs. The first clinical case included medication review for a person taking five or more medicines and discussed how the GP and pharmacist might jointly help address this patient’s ongoing care with particular reference to questions around polypharmacy with a pharmacist. The second clinical case was about a patient requiring an emergency supply of their medication and discussed how the pharmacist might approach this question and how the patient`s GP may be involved in deciding the outcome. The IPE students received a 3-h long session in total composed of (i) 30-min introductory lecture about the importance of prescribing in primary care (ii) study of each case study for 30 min and (iii) the rest of IPE session was dedicated to interactive discussions with teachers and fellow learners.

**Fig. 1:**
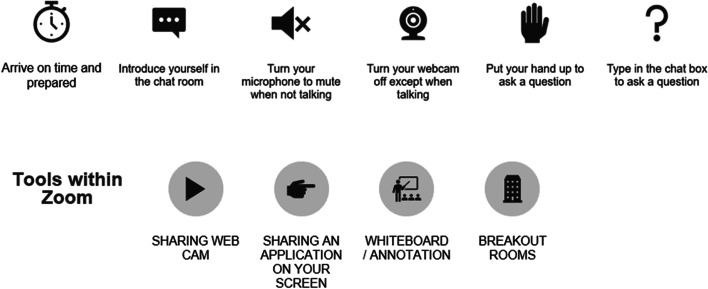
A slide explaining the online etiquette students are expected to adhere to in the synchronous IPE session

The first case study was completed individually albeit synchronously so students could observe how their peers responded in real time. The second case study was completed collaboratively in breakout rooms between pharmacy and medical students. Tutor notes were prepared by GPs and pharmacists together to provide consistency in delivery across the sessions. Feedback sessions were run jointly thus modelling interprofessional collaboration. A slide on “Zoom etiquette” was included to ensure the smooth running of the session; this is shown in Fig. 1. Attendance at an IPE session was compulsory for pharmacy and medical students.

Tools used included Zoom [21] to run the sessions and Mentimeter [22] to allow students to answer live clinical questions in a voting format, whereby results were instantly accessible to all participants.

### Study size

Up to a total of eight medical and pharmacy students were allocated per breakout room; 11 breakout rooms were set up in each session and the six facilitators rotated between the rooms to answer questions within the case study, if needed.

### Evaluation survey

At the end of the IPE session, students were asked to complete a bespoke feedback survey. The questions were designed by agreement between the sessions leads. The survey was purposefully a bespoke design rather than based on published literature to enable the collection of students` perceptions and suggestions in-depth via open ended questions as well as understanding their choice of teaching delivery route of IPE. The design of the survey was based on a multi-method study that collected both qualitative and quantitative information. Data collection was via Qualtrics software [23].

The survey consisted of 10 multiple-choice questions (Table 1) and a final three open-ended qualitative questions. The first nine questions presented statements with a 5-point Likert scale response. Ordinal data were converted to numeral values as follows:

- Strongly agree = 1;
- Somewhat agree = 2;
- Neither agree nor disagree = 3;
- Somewhat disagree = 4;
- Strongly disagree = 5.

**Table 1:** A table showing the questions asked and the percentage and absolute numbers of answers for each response (n), total (N). *P*-value represents statistical difference between pharmacy and medical students. Significance level was set at *P* < 0.05

	**Question**	**Strongly agree** **n (%)**	**Somewhat agree** **n (%)**	**Neither agree nor disagree** **n (%)**	**Somewhat disagree** **n (%)**	**Strongly disagree** **n (%)**	***p*-value**
1	I have found this IPE session has provided me with the opportunity to explore the concept of prescribing in primary care from another professions' perspective **(*N* = 97)**	52 (54)	38 (39)	6 (6)	1 (1)	0 (0)	0.0143
2	I have gained an appreciation of the importance of including service users in prescribing decisions **(*N* = 97)**	44 (46)	41 (42)	7 (7)	4 (4)	1 (1)	0.1669
3	Overall, I found the IPE session aims were made clear at the start of the session **(*N* = 97)**	48 (50)	36 (37)	10 (10)	3 (3)	9 (0)	0.4560
4	I would recommend this IPE session to other students **(*N* = 96)**	48 (50)	30 (31)	14 (15)	3 (3)	1 (1)	0.8768
5	This IPE session ran smoothly and was well organised **(*N* = 97)**	61 (63)	27 (28)	5 (5)	4 (4)	0 (0)	0.3029
6	I found the instructions provided during the Webinar easy to follow **(*N* = 97)**	52 (54)	30 (31)	9 (9)	5(5)	1 (1)	0.8436
7	I found the amount of information provided before the Webinar contained just the right amount of information **(*N* = 97)**	36 (37)	43 (45)	7 (7)	8 (8)	3 (3)	0.7252
8	I have found this IPE session has provided me with opportunities to appreciate the role of pharmacists in prescribing in primary care (*answered by medical students only*) **(*N* = 32)**	23 (72)	8 (25)	1 (3)	0 (0)	0 (0)	NA
9	I have found this IPE session has provided me with opportunities to appreciate the role of GPs in prescribing in primary care (*answered by pharmacy students only*) **(*N* = 59)**	22 (37)	31 (53)	5 (8)	1 (2)	0 (0)	NA

Question ten asked about the preferred mode of session delivery. The students were also asked to select if they are a pharmacy or medical student.

The open ended, qualitative response questions were:

- What was the most useful thing you learnt from the IPE session?
- Was there any additional content that you would have liked to have included in the session?
- Do you have any other comments that would help us to improve this session for future students?

### Statistical methods

Data were analysed using Origin Pro 2019 [24]. The full dataset was subject to a normality test to determine whether parametric or non-parametric statistical analysis would be most appropriate. The Shapiro–Wilk test rejected normality of the dataset, therefore non-parametric statistical analysis was undertaken. Since the data were non-parametric, the Mann–Whitney test was used via GraphPad Prism 7.03 (GraphPad Software Inc., La Jolla, CA, USA). This test was used to derive statistical difference between the responses given by the pharmacy and the medical students; significance level was determined by *p*-value < 0.05. Data was also displayed as odds, which was calculated as the number of students that selected a response divided by the number of students that didn’t. Odds ratio is calculated by dividing the odds of a group divided by the odds of another group, for example when comparing pharmacy and medical student responses**.** In some cases, some questions were left unanswered, these data were excluded from the statistical analysis and the total number of responses was reduced accordingly. Students comments that were mentioned three times or more were listed in the results sections, and concepts were reported illustrating areas requiring improvement. This was analysed independently by two co-authors of this study.

## Results

### Participants

Four sessions were delivered to accommodate the large student number; an overall response rate of 29% (100/350) was achieved. The response rate for pharmacy students was 36% (65/183) and for medical students was 21% (35/167).

### Descriptive data

The questions and results summary are shown in Table 1. The odds of pharmacy students choosing somewhat agree or strongly agree for question 1 were 30% lower than for medical students, a statistically significant difference. Overall, 93% (90/97) of students that responded selected agree or strongly agree, for this first question, indicating that both professions have expanded their knowledge in that remit from the other professions’ perspective.

The 2nd question which explored the shared decision making of prescribing, was rated higher again by medical students with a median of 1 (strongly agree) as opposed to 2 (somewhat agree) for pharmacy students, but this difference was not statistically significant.

The odds of pharmacy students selecting agree or strongly agree for the third question, which explored whether the aims of the IPE session were made clear at the start, were 15% higher than medical students, demonstrating their appreciation of the session’s aims.

For question 4, which asked about recommending the session to others, medical students gave a median answer of 1 (strongly agree) whereas pharmacy students gave a median answer of 1.5; this difference was not statistically significant (*p*-value = 0.8768), and overall indicates that both student groups were satisfied with the session.

Questions five, six, and seven, explored whether the IPE session was well organised, had easy to follow instructions, and the amount of information was appropriate, respectively. Both pharmacy and medical students gave the same median responses: 1, 1, (strongly agree) and 2 (somewhat agree). Overall, this indicates that the sessions were perceived by the students as being well organised.

Pharmacy students were asked whether the session helped them appreciate GP roles more, and vice versa. The median response for medical students was 1, or strongly agreeing with the statement that the IPE session helped in appreciating the pharmacist’s various roles in primary care prescribing.

The median response for pharmacy students was 2, or somewhat agreeing with the statement that the IPE session helped in appreciating the GP’s role in primary care prescribing. 90% (53/59) of the students selected strongly or somewhat agree.

### IPE session delivery method

Table 2 shows the results of the students’ preference for IPE sessions mode of delivery. 39% (38/97) of the students opted to attend future IPE sessions in the same mode they received this current session (i.e. a synchronous online session). 15% (15/97) of the students selected asynchronous online session (pre-recorded session) as their preferred mode of delivery. The remaining students selected face-to-face live sessions as their preferred method of delivery. Overall, 53% (33/62) of pharmacy students and 57% (20/35) of medical students preferred a remote method of delivery. These data are in close agreement with one another and demonstrate that remote delivery is acceptable for the facilitation of future healthcare IPE sessions, if appropriate.

**Table 2:** Showing the mode of delivery preference of pharmacy and medical students

Method of delivery	Pharmacy n (%) out of 62	Medicine n (%) out of 35	Total n (%) out of 97
Synchronous online session	22 (35)	16 (46)	38 (39)
Asynchronous online session	11 (18)	4 (11)	15 (16)
Face to face	29 (47)	15 (43)	44 (45)

### Qualitative findings

When asked about the most important thing the students learned it was found that pharmacy students valued the “GP viewpoint” as the most useful learning from the IPE session, followed closely by [rules on] emergency supply of controlled drugs (CDs). This is in close agreement with medical students’ perspective that rated “CD rules” as the most valuable topic learnt in the sessions, followed by the difference between Summary Care Records (SCR) and Patient Medication Records (PMR) and the role of the pharmacist. These findings indicate that the IPE learning objectives of healthcare professionals learning about each other’s role remits had been met.

When asked about additional content to add in future IPE events, students focused on areas such as adding pre-work, more in-depth review of the other profession, more detailed feedback at the end of the sessions and increasing the number of facilitators. Specific examples of the student’s qualitative responses included the following:

#### Pharmacy students’ responses:

- Explanation more of the medical route from F1 to consultant
- Being given prework before session to focus knowledge
- A summary slide at the end of the IPE session
- More scenarios that would be aimed at teaching pharmacists what the GP role is, rather than the reverse.

#### Medical students’ responses:

- Uploading slides ahead of the day and including the schedule of the day
- More in-depth review of the pharmacist role
- More in-depth review on CD prescribing and prescription requirements
- Would prefer to discuss answer to each question to live poll immediately, rather than all at the end (referring to case 1)

When asked if the students have any additional comments, the responses were as follows:

#### Pharmacy students’ responses:

- More time for feedback and discussion
- More detailed explanations
- Give prework
- Access to resources prior to session

#### Medical students’ response:

- Best to have right or wrong choices, instead of ranking, as not realistic in real life
- A staff member to remain in breakout room to encourage participation
- Ensuring time is adequate for the exercise
- Wasn’t clear that each group was numbered, so when feedback came it was confusing

## Discussion

### Key results

Pre-registration IPE is a powerful tool to prepare healthcare professionals for the expectations of working in a multi-disciplinary team and to maximise patient care [25, 26]. The choice of prescribing as the theme for this IPE session was derived from the fact that the topic is shared and collaborative between these professions in the real world [27]. The findings of this research have suggested that medical students, as compared to pharmacy students, felt the session provided them with a better understanding of pharmacists’ roles in prescribing in primary care. This is in line with a US-based study found that an IPE activity between pharmacy and medical students in a clinical setting increased the understanding of both professions roles as well as their own roles [28]. Dispensing medicines and ensuring prescriptions are legally and clinically valid are essential services for pharmacists under the NHS contractual framework [29] and therefore in the core remit of a pharmacist’s role. This may indicate that pharmacy students are more likely than medical students to be familiar with recent legislation such as that surrounding controlled drugs, and thus this may explain why medical students found the session more useful in that respect. Medical students had a higher agreement rate that the content regarding including patients in shared decision making was found to be more useful. 87% (84/97) of the students that completed the survey selected agree or strongly agree for finding the aims of the session clear, indicating that the instructions provided were sufficient, albeit with some room for improvement. Similarly, 81% (79/97) of students selected agree or strongly agree for recommending the session to others, which re-iterates the finding that the student improved their learning as a result of this IPE activity. This is in line with studies which identified that, during IPE sessions, healthcare students reporting working as a team and with other healthcare professionals is important [30]. Pharmacists and doctors share factual competencies as well as professional ones, that can be ideally placed in an IPE event [31].

With regards to the question relating to the organisation of the session, it was found that the odds of students that responded choosing to agree or strongly agree were 10 times higher than choosing the other responses, demonstrating their appreciation of the session’s organisation. These findings may demonstrate that the learning outcomes achieved were aligned with the objectives set, an important parameter in traditional pedagogy and in IPE learning [32]. Overall the most selected answer, or the mode, by students who responded was strongly agree for questions 1 to 6 and somewhat agree for question 7. This demonstrates that most of the participants felt like the amount of information provided was either higher or lower than necessary. This is an issue that will need to be explored further, possibly in focus groups, to improve future IPE activities.

### Interpretation

A key goal of IPE activities is to evaluate whether the objectives have been met [33]. In this activity, it is evident that students from both professions have benefitted and successfully made improvements in their awareness as a result of the session. This is in line with a US-based study where an evaluation of pharmacy and medical students ability to collaborate inter-professionally has been increased by 79% (253/320) as a result of an IPE event [34].

Both groups have found the IPE session to be useful in learning about the other’s profession (Q8 and Q9), which is consistent with findings in other IPE sessions [35, 36]. However, the respondents have indicated that they would like to have a more in-depth review of the other profession’s role, more specifically pharmacy students indicated they would like to learn more about the role of the GP in the qualitative section of the survey. These findings are in line with literature reporting that healthcare students have limited exposure to other professions during their training, which may affect their ability to collaborate in the longer term [37]. Training with other professions has also been found to improve sense of usefulness and to have clarity in extend of one’s role in a collaborative team [38].

In an era of digital healthcare and telemedicine, it is imperative to build expertise in the use of online resources in undergraduate curricula, and in particular in IPE, where remote interaction between healthcare professionals has significant potential benefits [39]. A study that used a virtual environment to conduct an IPE activity for healthcare students from different cities, has indicated the students found the platform easy to use and that it facilitated interaction and learning remotely [40]. Over half of the participants selected a remote method of delivery as their preferred method of delivery for this IPE event. These positive results may be due to the IPE session’s technological success, as the sessions were aided through the use breakout rooms, chat functions and having a technical lead available; all tips of success as shared by a recent review of synchronous IPE sessions [41]. Another study that evaluated the perception of pharmacy and medical students working collaboratively on a telehealth project, indicated similar findings to our study, where students from both professions indicated an improved appreciation of the other even though the IPE event was ran remotely [42].

The data gathered demonstrate that students valued prescribing information gained as well as soft skills such as communication and teamwork, both essential for work in the healthcare services [43]. These themes are consistent with common IPE learning themes as identified by a recent UK mapping study [3]. When asked about additional content to be included, the findings between both student bodies are similar, with a focus on ensuring the availability of pre-work. In this activity, there was no pre-work assigned. The final qualitative question asked about improvements for the future. In this instance, there was approximately one facilitator per two breakout rooms. Students indicated that they needed more time for feedback and more detailed explanations. This is in line with evidence that suggests that facilitators help students synthesise their learning, translate learning problems into learning opportunities, and indeed resolve conflict between students, if applicable [44]. It is to be noted that facilitation of students from different disciplines has been acknowledged as a difficulty and can be aided with operational support [45]. In addition, it may be challenging to find willing faculty members to participate in the events, as it can be seen as an extra workload; leaders have a responsibility here to motivate academic members [46]. The other main issue mentioned repeatedly by students is the availability of pre-work and posting clear instruction beforehand. Evidence in the literature suggests that the assignment of pre-work aids in the understanding during teaching sessions and thus this is something to be considered for future IPE activities [41].

### Limitations

Overall, this IPE activity included a large student number, albeit the participation was low where only 100 out of 345 students completed the survey (29%). Due to this low response rate, the findings may not be fully representative of the student body and the reasons for not taking up the survey should be fully explored in future studies. A weakness of this study is that it is held only in a single higher education institute, and that there was a disproportionate ratio between the respondents from the pharmacy and medical students, thereby potentially skewing the total results towards the opinion of pharmacy students. Another limitation of this study is that the evaluation consisted of student perceptions of learning, which is limited by the design of the questions, and by the students themselves Additionally, the responses were collected shortly after the end of the sessions, and it has been suggested that measuring timepoints in a more distant future, for example one year, may give a more reliable view of the long-lasting effects of the IPE event and whether the answers remain consistent [47]. It is to be noted that a further limitation may be that this survey only measured the students’ ‘reaction’ level of Kirkpatrick’s hierarchy, used for evaluation of training and learning [48]. All these issues can be explored in future studies.

### Generalisability

Overall, this study has contributed to the literature by showing that (i) remote delivery of IPE in an interactive format was accepted by some students; hence considering the logistical challenges often associated with IPE delivery in terms of room booking and timetabling can be overcome by implementing online IPE delivery when needed; (ii) the content of IPE should be well-balanced in terms of selected topics as this enables multiple health professions programme students to allow them contributing to case study discussions equally, hence learn from each other and about their roles in future professional practice and (iii) bespoke survey used may influence the design of new questions for relevant qualitative methodologies.

## Conclusions

This paper demonstrates that the IPE sessions described have proven helpful for both medical and pharmacy students in terms of increasing their understanding of prescribing principles and developing their teamwork and communication skills. This could indicate the that IPE education will positively impact the practice of future doctors and pharmacists as these simulations prepare them for real-life practice. The findings of this study suggest a practical way for future planning of IPE sessions, where the use of blended learning approaches can be promoted. This includes combining of face-to-face and remote delivery, which encompasses both synchronous and asynchronous online sessions. In addition, students from both courses indicated that they have gained a deeper understanding of the other profession with a focus on prescribing in primary care which could pave the way to incorporating IPE learning from earlier stages in these healthcare courses. This study has highlighted areas of improvement for future sessions, in particular availability of the sessions` schedule beforehand as well as assigning pre-work, and the availability of a sole facilitator per breakout room / face-to-face group.

## Data Availability

All data generated or analysed during this study are included in this published article.

## Abbreviations

IPE: Interprofessional education
GP: General practitioner
GMC: General Medical Council
GPhC: General Pharmaceutical Council
NHS: National Health Service
UCL: University College London
UK: United Kingdom
US: United States
QR: Quick Recognition
